# Development of Teachers’ Emotional Adjustment Performance Regarding Their Perception of Emotional Experience and Job Satisfaction During Regular School Operations, the First and the Second School Lockdown in Austria

**DOI:** 10.3389/fpsyg.2021.702606

**Published:** 2021-11-15

**Authors:** Katharina-Theresa Lindner, Hannu Savolainen, Susanne Schwab

**Affiliations:** ^1^Department of Education, University of Vienna, Vienna, Austria; ^2^Department of Education, University of Jyväskylä, Jyväskylä, Finland; ^3^Center for Teacher Education, University of Vienna, Vienna, Austria; ^4^North-West University, Vanderbijlpark, South Africa

**Keywords:** COVID-19, teachers’ emotional experience, job satisfaction, longitudinal study, school lockdown

## Abstract

Starting with the COVID-19 pandemic, research intensively investigated the effects of school lockdowns on involved stakeholders, such as teachers, students and parents. However, as research projects had to be hurriedly conducted, in-depth and longitudinal studies are lacking. Therefore, the current study uses data from a longitudinal study to investigate the well-being of Austrian in-service teachers during the COVID-19 pandemic. In total 256 teachers took part at both measurement waves and participated in an online survey. Standardized questionnaires were used to assess teachers’ perception of emotional experiences and job satisfaction before COVID-19 (retrospective, t1), during the first (*in situ*, t2) and during the second school lockdown (*in situ*, t3). The results indicated that the vast majority of teachers generally felt a high level of job satisfaction. However, teachers’ satisfaction decreased between regular teaching and school lockdowns. Similarly, positive emotional activation was reduced and negative activation increased. Further, results from a positive activation cross-lagged path model indicated that the lack of positive activation led to lower job satisfaction. For negative emotional activation, job satisfaction during the first school lockdown predicted negative activation at the second lockdown.

## Introduction

Due to the spread of the COVID-19 virus, educational processes and regular school operations have been turned upside down. Because of the worldwide increase in infections and the unpredictability of the ongoing pandemic, new basic conditions for teaching and learning processes needed to be established and implemented in a short amount of time (Helm et al., 2021; Lindner et al., 2021). In the context of a constant transition between classroom and remote schooling, not only aspects of teaching and learning underwent change, but also factors on the individual level were influenced by countless upheavals due to new circumstances and associated challenges (Dabrowski, 2020).

In this context, intrapersonal characteristics on the teacher level, which are considered to influence schooling and education during regular school operation, need to be investigated under the novel conditions of COVID-19. For this reason, job satisfaction and teachers’ emotional experiences were examined before and throughout the development of the pandemic to investigate their emotional adjustment under these extraordinary conditions. Teacher attrition and rising dropout rates of those who leave the profession can often be traced back to low job satisfaction (e.g., Heikonen et al., 2017) and the lack of emotional well-being of teachers (e.g., Van Horn et al., 2004; Hong, 2012).

According to Heller et al. (2002), job satisfaction is defined as the fulfilment an individual perceives through performing work that is associated with their job. It also includes the emotional state of satisfaction associated with the job regarding not only in the performance of the job itself but also at an abstract psychological level by considering how employees think or feel about the job. Therefore, fulfilled job satisfaction is realized by the positive attraction an individual has toward their job. This is influenced by intrapersonal factors, such as values, expectations, and beliefs (Heller et al., 2002; Alonso, 2006; Drafke, 2009). As can be seen from the working definition of job satisfaction, the emotional experience of teachers plays a significant role in this context. For this purpose, the current study also focuses explicitly on teachers’ emotional experience before and during the COVID-19 pandemic by taking into account the positive and negative affective activation of teachers.

The concept of positive activation (PA) and negative activation (NA) encompasses the idea of affective activation systems that constitute the personality of individuals (Watson and Tellegen, 1985; Schreiber and Jenny, 2020). The core concept of PA/NA builds on the idea of comprising high PA by positively valued affective states with a high degree of activation (e.g., wide awake), whereas negatively valued affective states are at the low level of PA (e.g., tired). The same concept applies in reverse for NA; high NA encompasses negatively associated affective states with a high level of activation (e.g., worry), with its counterpart, “free of worries” at the low end of the positively associated affective state regarding a low NA (Schreiber and Jenny, 2020). Therefore, whether an affective dimension is considered to conceptualize PA or NA depends on the high level of activation of its affective status. In other words, PA indicates a high activation state considered to be a positive affect, whereas NA indicates a high activation state considered to be a negative affect.

### Teachers’ Job Satisfaction and Emotional Experience Before COVID-19

#### Job Satisfaction

Teachers’ job satisfaction and emotional experience are often considered to be significant factors regarding the quality of teaching processes and pedagogical professionalism. Regarding predictors of teachers’ job satisfaction, existing research demonstrates diffused insights into the relation between gender and teachers’ job satisfaction. Some studies indicate higher levels of job satisfaction for female teachers (Liu and Ramsey, 2008; Toropova et al., 2021), while other studies highlight the same outcome for male teachers (Klassen and Chiu, 2010; Aydin et al., 2012). Still other studies do not report gender as a predictor for teachers’ perception of job satisfaction (e.g., Eliophotou Menon and Athanasoula-Reppa, 2011).

Research on the relation between teaching experience and job satisfaction has also shown diverse outcomes. Some studies report a positive correlation of years of teaching experience and job satisfaction (e.g., Eliophotou Menon and Athanasoula-Reppa, 2011), whereas others show a U-shape curve, highlighting novice and expert teachers as having higher levels of job satisfaction than mid-experienced teachers (Crossman and Harris, 2006). In contrast, a meta-analysis considering data from a United States context (Guarino et al., 2006) and a study on Canadian teachers (Klassen and Chiu, 2010) indicate that especially low- and high-experienced teachers tend to quit their teaching jobs due to low job satisfaction levels.

Regarding the occupation level of teachers, i.e., whether they are teaching at a primary or secondary school level, research indicates that secondary teachers report significantly higher scores on job satisfaction than primary school teachers (e.g., Sharma and Jyoti, 2006). In contrast with this assumption, results of Allen (2005) show higher dissatisfaction levels for secondary school teachers.

#### Emotional Experience

Teacher well-being is considered a key factor for successful schooling and predicts teachers’ choices about leaving the profession (Naghieh et al., 2015). According to teachers’ emotional experiences during work, several studies show that, in general, the teaching profession is associated with high levels of stress (Stoeber and Rennert, 2008; Liu and Onwuegbuzie, 2012; Skaalvik and Skaalvik, 2015; Fitchett et al., 2018). In regard to job-related stress in terms of workload, results of previous studies show that women perceive significantly higher stress levels than their male colleagues (Klassen and Chiu, 2010; Nasser-Abu Alhija, 2015). The degree of stress caused by workload and significant differences were found between primary and secondary school teachers. Results of the study of Nasser-Abu Alhija (2015) reported higher stress levels for primary teachers compared to their counterparts in secondary schools. Years of work experience showed a non-linear relation to stress caused by workload and classroom stress increasing from novice teachers to mid-experienced teachers, but decreasing among those with long-term teaching experience (Klassen and Chiu, 2010).

Regarding teachers’ perception of energy and exhaustion, studies show that female teachers report significantly higher levels of emotional exhaustion (Skaalvik and Skaalvik, 2011, 2017; Rumschlag, 2017). Rumschlag (2017) indicated a significant difference between novice (less than 5 years) and expert (5 years and longer) teachers regarding the emotional experience of exhaustion. Tsigilis et al. (2011) examined potential burnout factors and emotional exhaustion among physical education teachers in primary and secondary schools. Due to work circumstances and conditions (e.g., higher intrinsic need for mobility among primary school students) and associated professional demands for teachers, results show higher levels of emotional exhaustion in the sample of secondary school teachers (Tsigilis et al., 2011).

By investigating indicators of teachers’ professional identity (including job satisfaction and job motivation of teachers), no significant differences between novice teachers and expert teachers were found by Canrinus et al. (2012). Additional research on gender differences indicated a higher level of motivation among males than their female colleagues (Bishay, 1996). Other studies showed no significant differences between gender and job motivation (Dieleman et al., 2003; Lim, 2014; Nagrath, 2019). In a study conducted by Kunter et al. (2008), no correlation between teachers’ enthusiasm and individual characteristics, such as gender and teaching experience, were found.

#### Relation Between Teachers’ Job Satisfaction and Emotional Experience

Previous research on teachers’ job satisfaction and its impact on individual factors indicated that it contributed to teachers’ emotional well-being, or, in other words, job satisfaction correlates negatively with somatic symptoms such as stress and burnout (Cheryl and Cooper, 1993; Chaplain, 1995; Ho and Au, 2006; Klassen et al., 2010; Skaalvik and Skaalvik, 2011; Toropova et al., 2021). In a study by Kunter et al. (2008) a moderate relation of teachers’ job satisfaction and general self-reported job enthusiasm was found.

### Education Conditions During the COVID-19 Pandemic in Austria

The COVID-19 global pandemic caused all-encompassing challenges and changes to daily life in Austria. First school closures were initiated during the first lockdown of public life on March 16, 2020. As a consequence, regular school operations shifted from face-to-face lessons to remote learning, which mostly resulted in synchronous and asynchronous digital teaching and learning for teachers and students at all educational stages. Higher secondary schools were closed on March 16, and primary and secondary schools closed 2 days later. For parents who had to work and/or were not able to take care of their children during homeschooling, day care at school was provided (BMBWF, 2020b).

After 2 months of remote schooling, Austrian schools reopened for in-class teaching. First the graduating classes of higher secondary school opened, followed by the primary and lower secondary schools (BMBWF, 2020a). Due to the increase in the number of COVID-19 cases, regular school operations were again changed to home schooling throughout Austria. The second nation-wide lockdown started with the closure of higher secondary schools (starting at Grade 9) on November 3, 2020, followed by all other school types on December 4 (BMBWF, 2020c). In-between these two phases of nationwide school lockdowns, schools were attended in shifts by students. One group attended on Monday and Tuesday, and the other group on Wednesday and Thursday, while remote learning was conducted on other days of the week (BMBWF, 2020d).

### COVID-19 and Its Implications for Teachers’ Perception of Job Satisfaction and Emotional Experience

Huber et al. (2020) highlighted that approximately 40% of teachers (*N* = 1,676) felt (rather) burdened at the beginning of the pandemic (end of March 2020).

The results of Schwab et al. (submitted^1^) indicated that 60.8% of Austrian teachers (*N* = 3,467) felt a high potential for professional burden during the first lockdown of the schools. The percentage of teachers who reported being (rather) concerned during the first school closures in Austria was 46.6%.

In the study of Mikušková and Verešová (2020), 379 Slovakian teachers were asked among other things about their emotional state and job satisfaction during the pandemic. Results showed that primary school teachers’ positive emotions correlated positively with their job satisfaction operationalized by their satisfaction with institutional support. In addition to this finding, decreasing positive emotions were related to lower satisfaction with institutional support and the increased teaching of topics with which students were already familiar. For the sample of higher secondary teachers, similar results were found. Negative emotions correlated negatively with teachers’ job satisfaction regarding institutional support (Mikušková and Verešová, 2020).

A study of Vietnamese teachers’ experience (*N* = 294) during the COVID-19 pandemic provides interesting insights into teachers’ job satisfaction. Descriptive results showed that female teachers reported higher satisfaction than male teachers. Regarding the level of teaching experience of teachers as a predictor of job satisfaction, the outcomes showed that novice teachers (less than 3 years of experience) and expert teachers (more than 10 years of experience) reported similar levels of job satisfaction reaching higher levels than those teachers who had an in-between work experience of 3–10 years. The highest levels of job satisfaction were reported by higher secondary teachers, followed by primary teachers and lower secondary teachers (Vu et al., 2020).

Additionally, the two related factors were associated with a lower perception of somatic burden, stress, and emotional exhaustion (*N* = 325 Australian teachers; Collie, 2021).

Highlighting the importance of longitudinal studies and results regarding the impact of dramatic and fundamental changes due to the spread of COVID-19, Sokal et al. (2020) provided insights into important findings regarding teachers’ development throughout the first 3 months of the pandemic. By asking 1,626 Canadian teachers about their attitudes toward change, efficacy, and burnout during the pandemic, the participants reported increased exhaustion and lack of energy, but simultaneously perceived increased efficacy in handling classroom processes. However, the participating teachers demonstrated increased negative cognitive and emotional attitudes toward change throughout the 3 months of study (Sokal et al., 2020).

Letzel et al. (2020)’s longitudinal study (*N* = 124 German teachers) (2021) indicated a significant increase of German teachers’ NA regarding the transition from regular school operation to remote education, whereas for the same period, a decrease of teachers’ PA was observed. More precisely, teachers’ emotional experience during homeschooling regarding the feelings of being angry, nervous, worried, and bored increased compared to regular school operation before the pandemic.

Alves et al. (2021) conducted a study among 1,479 Portuguese teachers asking them about their well-being before and during the pandemic and its associated remote teaching. Regarding their job satisfaction, interesting findings were presented comparing their perceptions before and during the pandemic. Prior to the pandemic, teachers with less than 10 years’ experience were more satisfied than teachers with more years of experience. In addition, before the pandemic, teachers at lower school levels were more satisfied with their job and the education system than those in secondary schools (Alves et al., 2021). Comparing these findings with teachers’ answers regarding their job satisfaction during the pandemic, the results draw the following picture: Being female, having a service time of less than 20 years, increasing well-being, decreasing perceptions about teaching difficulties and increasing positive future perspectives, contribute to the increase in positive perceptions of professional well-being.” By taking local regions of Portugal into account, the results differed in some ways (Alves et al., 2021).

### This Study

Against the background of newly arisen professional challenges due to COVID-19 and associated school lockdowns, this study investigates Austrian teachers’ development of job satisfaction and emotional experience regarding their PA and NA from regular school operations to recurring school closures. By examining teachers’ perceptions referring to different measurement points (regular school operation before COVID-19, first Austrian school lockdown from March to May 2020, second Austrian school lockdown from November to December 2020) the manuscript allows for insights into teachers’ emotional adjustments. School operations during COVID-19 are explored from a longitudinal perspective. What is not yet clear is the influence of the changed basic conditions on teachers’ perceptions of job satisfaction and emotional experience, two decisive factors for professional action. Due to the novelty and continuing topicality of education under global circumstances of COVID-19, longitudinal data about educational processes and the individual development of involved stakeholders are limited. Therefore, this manuscript critically discusses the following research questions:

(1) Are there changes in Austrian teachers’ job satisfaction and emotional experience between regular school operations (t1), first school lockdown (t2) and second lockdown (3) due to COVID-19?
(2) Are teachers’ gender, expertise (novice or expert), or maintaining their position in elementary or secondary education related to changes in job satisfaction and emotional experience?
(3) Does job satisfaction later predict PA of teachers or vice versa?
(4) Does job satisfaction later predict NA of teachers or vice versa?

In light of the research objectives and research questions, this study makes a major contribution to research on teachers’ emotional adjustment in the context of schooling during the COVID-19 pandemic in Austria by presenting results of a longitudinal study.

## Materials and Methods

### Participants

The sample of the current study was derived from two measurement points of the INCL LEA study. The first measurement point (t1) took place during the first lockdown of schools from March to May 2020, in the course of which 3,467 Austrian teachers (2,839 female, 14 diverse; 36.3% from primary schools and 40% from secondary schools). Participants represented all nine federal states of Austria in an online survey on homeschooling to gain insight into teachers’ stress level before and during the homeschooling situation. The second survey phase (t2) was carried out from November to December 2020. At t2, 2,651 Austrian teachers completed the online questionnaires (2,159 female, 7 diverse; 35.6% from primary schools and 31% from secondary schools) to provide insight into their emotional experience during the second school closure in Austria.

The current study focuses only on teachers that participated in the first and second surveys, as the methodological center of attention lies on the longitudinal development of teachers’ perceptions. Regarding only the longitudinal sample, 256 participating teachers were considered for this purpose, and the sample consists of 220 female teachers. The average age of teachers was 44.63 years (std = 11.60) and the average amount of teaching experience in years is 19.45 (std = 12.66). Following the suggestions of previous studies, the novice cutoff of teaching experience was 5 years (0–5 years novice; 6 years and over, expert). The participants included 49 novice teachers (19.1%) and 204 expert teachers (79.7%). Of this number, 42.2% taught in primary schools and 40.6% in secondary schools.

### Instruments

#### Job Satisfaction

The Enzmann and Kleiber (1989) scale was assessed to determine teachers’ job satisfaction. Six items were rated on a four-point Likert scale (1 = totally true to 4 = not true at all). The items were positively and negatively formulated to assess teachers’ satisfaction (e.g., “I enjoy my job.”) as well as dissatisfaction (e.g., “I have previously seriously considered quitting my job”). Results of previous studies show internal consistency of Cronbach’s alpha = 0.79 (Delgrande et al., 2005) and 0.72–0.79 in the Swiss context (Sandmeier et al., 2017). In the context of the current study, Cronbach’s alpha for job satisfaction regarding t1 = 0.72, at t2 = 0.78, and at t3 = 0.81.

#### Emotional Experience

The PANAVA Scale (Schallberger, 2005) was used to assess teachers’ emotional experience. This scale consists of eight items regarding affective experience states by asking participants about their emotional state in the context of opposite adjectives. These adjective pairs can be distinguished into dimensions of PA and NA. The pairs, for example, “full of energy” and “no energy,” were ranked on a rating scale with seven increments. In this context, positive and negative activating dimensions do not represent actual positive or negative affective states. For example, “full of energy” is an item that possesses high PA; however, its opposite, “no energy” is understood as an expression of low PA. One example item for NA is relaxed (low NA) and stressed (high NA; Schreiber and Jenny, 2020). Previous studies using the scale have shown acceptable internal consistency with Cronbach’s alpha = 0.89 (PA) and 0.86 (NA) (Zurbriggen and Venetz, 2018) and Cronbach’s alpha = 0.85 (PA) and 0.80 (NA) (Schreiber and Jenny, 2020). Considering the current sample, Cronbach’s alpha for PA was 0.81 (t1), 0.76 (t2), and 0.82 (t3); and for NA = 0.79 (t1), 0.80 (t2), and 0.77 (t3).

### Procedure

The current study was conducted within the COVID-19 related research project INCL LEA (Inclusive Home Learning during COVID-19) based at the University of Vienna. The research project was developed and implemented in cooperation with the local school boards in Austria, which also gave ethical approval for the study. Teachers from all federal states in Austria participated in an online survey during the first and second lockdowns of Austrian schools due to the COVID-19 pandemic (Kast et al., 2021; Lindner et al., submitted^2^; Schwab and Lindner, 2021; Schwab et al., submitted) (see text footnote 1). The collected data regarding teachers’ job satisfaction and emotional experience refers to three measurement points: t1 = regular school operation (teachers referred to t1 retrospectively during the first lockdown), t2 = first school lockdown in Austria (first simultaneous survey), and t3 = second school lockdown in Austria (second simultaneous survey).

### Analysis Strategy

Changes in the mean levels of the outcomes (Job satisfaction, Positive emotional experiences, and negative emotional experiences) were analyzed with repeated ANOVA measures with each outcome viewed separately. First a within time model with a single outcome in turn was estimated to test if changes between measurements in these outcomes were statistically significant. Within time contrast were used to test whether the possible change in outcome levels in time took place between t1 and t2 or between t2 and t3. Then models with each outcome as the within the time factor and the three background variables in turn as between level factors were estimated. Full models with within time main effect, within time interaction with each background group factor and the between groups main effects were calculated separately for each outcome with each background factor (RQ 1 and RQ 2).

**FIGURE 1:**
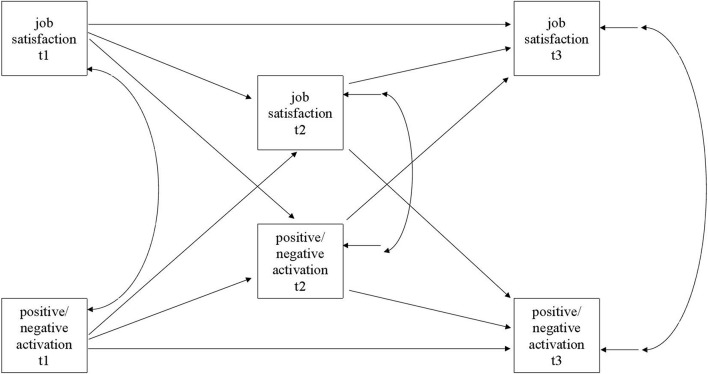
Theoretical path model.

Analyses of the direction of within time relationships between job satisfaction and the two other outcomes were analyzed using path modeling (Mplus 8, Muthén and Muthén, 2017). In each model a full model that contained all cross-lagged relationships, all stability paths, and correlations of simultaneous measures was estimated first (see theoretical model in Figure 1), and the model was then modified if the fit to the data was not adequate. Modification indices were utilized in making improvements to the model.

## Results

### Are There Changes in Austrian Teachers’ Job Satisfaction and Emotional Experience Between Regular School Operations (t1), First School Lockdown (t2) and Second Lockdown (t3) Due to COVID-19?

The descriptive results, intercorrelations, and reliabilities of the three outcome scales are shown in Table 1.

**TABLE 1:** Outcome scale correlations, reliabilities, means, and standard deviations.

*N* = 240–242	JS1	JS2	JS3	PA1	PA2	PA3	NA1	NA2	NA3	Cronbach alpha
JS1										0.72
JS2	0.39**									0.78
JS3	0.49**	0.48**								0.81
PA1	0.44**	0.05	0.24**							0.81
PA2	0.15*	0.49**	0.31**	0.09						0.76
PA3	0.18*	0.33**	0.56**	0.24**	0.52**					0.82
NA1	−0.36**	0.10	−0.19**	−0.60**	–0.06	–0.11				0.79
NA2	−0.13*	−0.46**	−0.23**	0.10	−0.52**	–0.19**	0.20**			0.80
NA3	−0.19**	−0.36**	−0.44**	−0.14*	−0.48**	–65**	0.27**	0.45**		0.77
Mean (std)	3.56 (0.40)	3.12 (0.56)	3.19 (0.59)	5.22 (1.03)	4.49 (1.06)	4.33 (1.21)	3.49 (1.13)	4.01 (1.28)	4.11 (1.22)	

JS1, job satisfaction at t1; JS2, job satisfaction at t2; JS3, job satisfaction at t3; PA1, positive activation at t1; PA2, positive activation at t2; PA3, positive activation at t3; NA1, negative activation at t1; NA2, negative activation at t2; NA3, negative activation at *t*. *p < 0.05, **p < 0.01.

Generally, Austrian teachers’ job satisfaction seems to be rather high, as all empirical mean scores are above three (on a four-point rating scale where 2.5 would be the theoretical mean of the scale). For job satisfaction, moderate correlations were found between the three time points (before lockdown, during the first and second lockdowns). For PA, however, no significant correlation was found between the time point before lockdown and during the first lockdown, but a low one was determined in the second lockdown. The correlation between the first and second lockdowns in PA was, however, moderate. For NA, the correlations of the time before lockdowns were small while those between the first and second lockdowns were moderate.

Results from ANOVA indicated that job satisfaction was reduced remarkably between the time of regular school operations and during the two lockdown measurements (see Table 2). While the overall within time effect was significant (*p* < 0.001) the within time contrasts indicated, however, that the difference was significant only between t1 and t2 (*p* < 0.001). Effect sizes were large (partial eta^2^ = 0.27 and 0.39). Positive emotional experiences also reduced during the studied period (*p* < 0.001; partial eta^2^ = 0.20), and contrasts indicated that that the change toward less positive emotional experiences was significant both between t1 and t2 and t2 and t3. The effect size of the initial drop was large (partial eta^2^ = 0.20), while the latter change had a small effect size (partial eta^2^ = 0.03). Negative emotional experiences increased significantly (*p* < 0.001; partial eta^2^ = 0.09), but the change took place only between t1 and t2 (*p* < 0.001; partial eta^2^ = 0.09). Effect sizes were moderate.

**TABLE 2:** Repeated measures analysis of variance results for all three outcomes.

	Within time	Time X group	Between groups	Within time effect contrasts		Time X group contrasts	
			
	*F* values and partial eta squared values			t1–t2	t2–t3	t1–t2	t2–t3
Job satisfaction (JS)	82.9*** (0.27)			145.6*** (0.39)	2.7 (0.01)		
JS and gender	26.5*** (0.11)	3.8* (0.02)	0.74 (0.00)	38.3*** (0.15)	0.15 (0.00)	6.7** (0.03)	4.2* (0.02)
JS and expertise	42.0*** (0.16)	3.4* (0.02)	0.54 (0.00)	70.4*** (0.24)	0.24 (0.00)	7.0** (0.03)	2.1 (0.01)
JS and position	70.9*** (0.27)	1.2 (0.01)	1.4 (0.01)	117.9*** (0.38)	0.93 (0.00)	2.2 (0.01)	1.2 (0.00)
Positive activation (PA)	56.2*** (0.20)			55.6** (0.20)	5.8* (0.03)		
PA and gender	19.9*** (0.08)	1.7 (0.01)	0.66 (0.00)	14.2*** (0.06)	6.5* (0.03)	3.0 (0.01)	1.5 (0.01)
PA and expertise	35.1*** (0.14)	1.1 (0.00)	0.23 (0.00)	28.7*** (0.11)	7.9** (0.03)	1.4 (0.01)	2.3 (–01)
PA and position	47.9*** (0.20)	0.72 (0.00)	1.4 (0.01)	52.3*** (0.21)	3.3 (0.02)	1.2 (0.01)	0.06 (0.00)
Negative activation (NA)	21.6*** (0.09)			21.0*** (0.09)	2.0 (0.01)		
NA and gender	7.0** (0.03)	0.52 (0.00)	0.60 (0.00)	6.0* (0.03)	1.1 (0.01)	0.76 (0.00)	0.02 (0.00)
NA and expertise	13.4*** (0.06)	0.08 (0.00)	0.61 (0.00)	12.8*** (0.05)	1.4 (0.01)	0.11 (0.00)	0.00 (0.00)
NA and position	17.6*** (0.08)	1.0 (0.01)	1.9 (0.01)	20.1*** (0.09)	0.44 (0.00)	0.88 (0.01)	0.19 (0.00)

eta^2^ values are partial eta squared values. *p < 0.05, **p < 0.01, ***p < 0.001.

### Are Teachers’ Gender, Expertise (Novice or Expert), or Maintaining a Position in Elementary or Secondary Education Related to Changes in Job Satisfaction and Emotional Experience?

**FIGURE 2:**
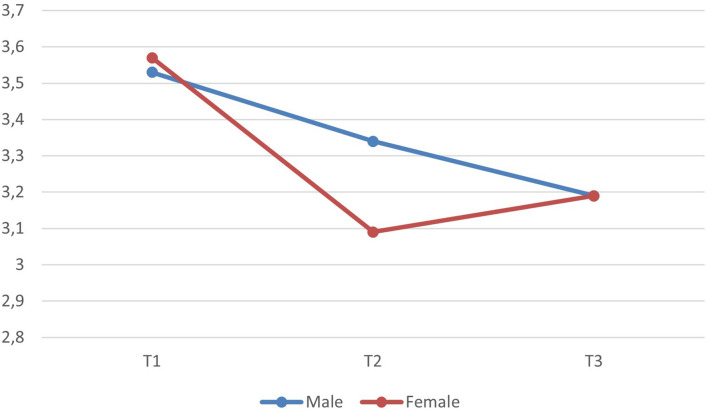
The relation of gender to development in job satisfaction.

Gender had a within time interaction effect (*p* < 0.05; partial eta^2^ = 0.02) indicating that the reduction in job satisfaction took place differently for males and females. Contrasts indicated that the gender effect was significant both between regular school operations (t1) and first lockdown (t2) (*p* < 0.01; partial eta^2^ = 0.03) and between t2 and t3 (*p* < 0.05; partial eta^2^ = 0.02). These interactions are depicted in Figure 2 and show that while male participants experienced a constant reduction in job satisfaction throughout the period, female participants experienced a significant reduction in job satisfaction between regular school operations and the initial lockdown but bounced back toward increased job satisfaction as they neared t3. There were no average between gender differences. Gender was not related to changes in positive or negative emotional experiences, nor were there any between gender differences in the average levels of these outcomes.

**FIGURE 3:**
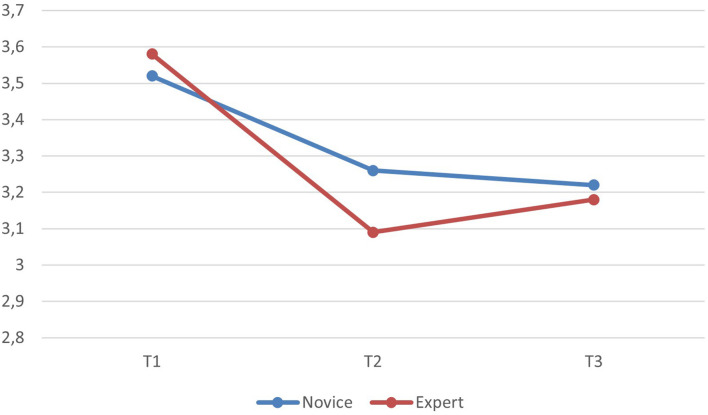
The relation of expertise to development in job satisfaction.

Expertise, i.e., comparison of novice and expert participants had a within time interaction effect to job satisfaction (*p* < 0.05; partial eta^2^ = 0.02), indicating that there were differences in how job satisfaction changed over the studied period in these groups (see Figure 3). The contrasts showed that the change in job satisfaction between t1 and t2 was different for novice and expert teachers (*p* < 0.01; partial eta^2^ = 0.03). Expert teachers seemed to react with a larger reduction in job satisfaction when the first lockdown took place, but their job satisfaction did not worsen as they approached t3. Novice teachers showed a milder drop in job satisfaction. There were no differences on the average in job satisfaction between these groups. Expertise was not related to changes in positive or negative emotional experiences, nor were there any differences on the average level of positive or negative emotional experiences between these groups.

The teachers’ position, i.e., whether they worked in primary schools or in secondary schools, had no effect on developments in any of the three outcomes. Neither were there any average differences on the level of job satisfaction, PA, or NA between primary school and secondary school teachers.

### Does Teachers’ Job Satisfaction Predict Later Positive Activation of Teachers or Vice Versa?

**FIGURE 4:**
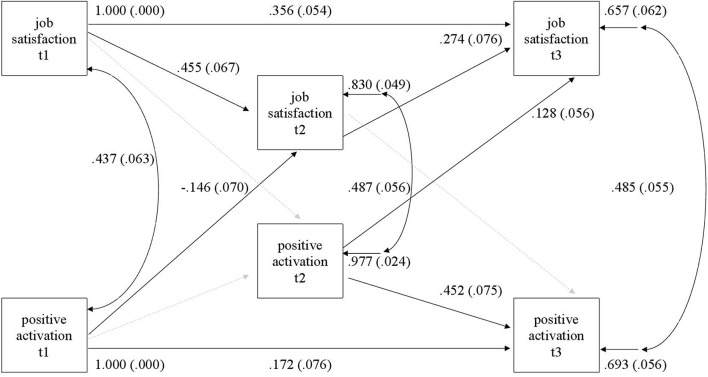
Structural equation model (SEM) of job satisfaction and positive activation. A dashed line indicates that the path is not statistically significant.

The model of job satisfaction and PA included all stability paths, correlations between same time outcomes, and all four cross-lagged paths. This initial model showed limited fit to data; therefore, an additional direct path between t1 job satisfaction and t3 job satisfaction and t1 and t3 PA were added to the model. The final model had a good fit to the data (chi-square = 1.21; df = 2; *p* = 0.54; CFI = 1.0; TLI = 1.0; RMSEA = 0.00; SRMR = 0.01). The final model is depicted in Figure 4, which shows only statistically significant paths; the non-significant paths were omitted, while they remained in the model.

The job satisfaction stability between t1 and t2 was moderate (Std.Beta = 0.46) and interestingly, t1 job satisfaction prediction of t3 job satisfaction seemed higher (Std.Beta = 0.36) than that between t2 and t3 (Std.Beta = 0.27). In contrast, PA showed no statistically significant stability between t1 and t2, but t1 activation was related to t3 activation (Std.Beta = 0.17). The cross lagged relationships were evident from PA to job satisfaction but not vice versa. Initial level PA predicted lower job satisfaction later during t2 (Std.Beta = –0.15).^3^ Positive activation in t2 predicted positive job satisfaction in t3 (Std.Beta = –13).

### Does Teachers’ Job Satisfaction Predict Later Negative Activation of Teachers or Vice Versa?

**FIGURE 5:**
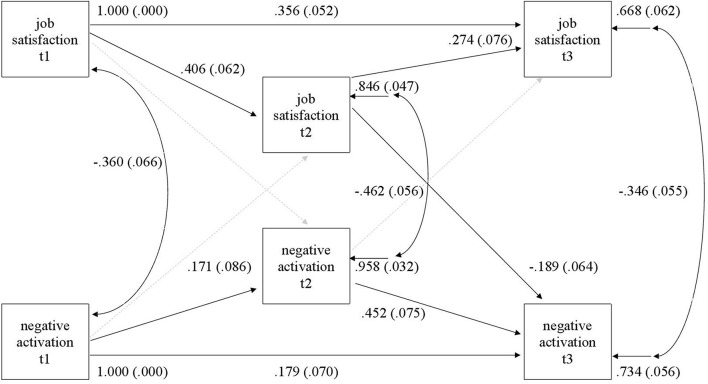
Structural equation model (SEM) model of job satisfaction and negative activation. A dashed line indicates that the path is not statistically significant.

The model with job satisfaction and NA included all stability paths, correlations between same time outcomes, and all four cross-lagged paths. After the initial test of fit, a direct path between t1 and t3 job satisfaction and between t1 and t3 NA were added to the model, which then fit the data very well (chi-square = 0.89; df = 2; *p* = 0.95; CFI = 1.0; TLI = 1.0; RMSEA = 0.00; SRMR = 0.00). The final model is depicted in Figure 5 which shows only statistically significant paths.

The stability of job satisfaction is similar to the earlier model. Negative activation had no significant stability between t1 and t2, but the direct path from t1 to t3 was significant (Std.Beta = 0.18), while stability between t2 and t3 was moderate (Std.Beta = 0.33). Simultaneous correlations between outcomes were all significant and negative, indicating that higher job satisfaction was related to less NA. Of all four cross-lagged paths, only a path from t2 job satisfaction to t3 NA was significant (Std.Beta =–0.19), indicating that the level of job satisfaction during t2 predicted lower NA in t3.

## Discussion

The current study presents a longitudinal approach that examines Austrian teachers’ job satisfaction and emotional experience before and during COVID-19 regarding three time-reference points. In the context of the discussion of the results, the phrase *time-reference points* instead of *measurement points* seems necessary for teachers’ perception at t1. The regular school operation data before COVID-19 was collected retrospectively at t2, a time where the spread of COVID-19 had already been declared a global pandemic and schools were closed for the first time in Austria.

This circumstance might explain the significant decrease of job satisfaction from regular school operations to the first closure of schools. As teachers were asked about their perception retrospectively, the uncertainty of the situation during the beginning of the pandemic and its associated new demands and challenges in the context of education and schooling might have had an impact on their view of times before COVID-19, in the sense of a glorification of the past. In this context, the results highlight a remarkably reduction of job satisfaction and PA when comparing regular school operation and the time during the two school lockdowns. Teachers’ NA increased significantly between t1 and t2. The results confirm the outcomes of other studies, such as Letzel et al. (2020); Sokal et al. (2020), and Alves et al. (2021).

Interesting results occur when comparing teacher differences in the perception of job satisfaction in regard to gender. While female teachers perceived a tremendous downfall in job satisfaction toward the initial lockdown, more than that of men, this negative trend was leveled and even turned slightly positive as they approached the second lockdown. At the same time male teachers perceived a more or less linear decrease in their job satisfaction, which ended at the same level as female teachers at t3.

One possible explanation might be the differences between private life and home circumstances of female and male teachers as to gender stereotypes and traditional gender role distributions. Females are still more likely to be associated with domestic work and taking care of children or elderly persons who need support. Following an Austrian study regarding home office and domestic work, results show that unpaid work at home (e.g., domestic work, taking care of underaged children) is primarily performed by females, “albeit a second parent is at home due to home office, unemployment or short-time work” (Mader et al., 2020). Against this background, the first tremendous drop of job satisfaction perceived by female teachers may be traced back to cumulating challenges and responsibilities that no one was prepared for, followed by adaptive performance and adjustment of understanding of job-related circumstances.

The results indicate that although the job satisfaction of female teachers significantly decreased initially compared to their male colleagues, they might be better able to adapt and adjust to the current circumstances as indicated by the end of decrease in job satisfaction. These results contradict the results of Vu et al. (2020) as well as Alves et al. (2021), who reported higher job satisfaction for female teachers. However, the adjustment performance of female teachers needs to be highlighted as a successful coping strategy that supports teachers’ improvement of job satisfaction during phases of crisis, such as the pandemic.

Regarding teachers’ years of experience in teaching, results showed a stronger decrease of job satisfaction for expert teachers from regular school operation to the first school lockdown than for novice teachers. However, in comparison to novice teachers, the development of expert teachers’ job satisfaction showed no additional drop, and the level of job satisfaction increased toward the second school lockdown. Novice teachers showed a milder drop in job satisfaction throughout the entire collection process. One explanation might be that expert teachers had developed successful teaching strategies that worked for them over the years, and they might have faced a stronger backlash throughout the pandemic, as they could no longer benefit from their regular teaching style and proven and successful didactic and pedagogical actions. Novice teachers are in an initial professional finding process, which includes testing different methods and approaches to fit students’ needs, and adaption to new circumstances might be easier for them.

Teachers’ emotional experience, gender, and years of experience were not related to PA or NA throughout the development of the pandemic. Neither was the teachers’ school level (primary or secondary school) a significant predictor of job satisfaction (in contrast with the results of Vu et al., 2020) or emotional experience. Therefore, it can be assumed, that the sudden change of institutional conditions and professional demands were perceived similarly, regardless of the educational level of teachers and the fact that affected pedagogues felt confronted with similar challenges. The outcome that none of the investigated variables predicted teachers’ emotional experience contradicts with previous studies on teachers’ well-being and PA and NA during COVID-19 (Mikušková and Verešová, 2020; Alves et al., 2021). As the PA decreased and the NA increased for all participating teachers similarly, regardless of the examined predictors (gender, teaching experience, school level), it can be assumed that the development of the spread of COVID-19 and the associated changes of the school system triggered similar emotional reaction among all participating teachers.

This can be explained, in part, by the lack of professional communication between the government, the local school authorities, school principals, and school staff. For instance, school principals and teachers received the information about modifications (e.g., school lockdowns, hygienic measures, etc.) via public press conferences. Therefore, teachers were not informed beforehand, and changes were often announced last minute (e.g., on Friday it was announced by the press that schools would be closed on Monday). Therefore, teachers were not prepared in time, e.g., they often lacked the technical equipment and prepared homework for students) (Helm et al., 2021; Kast et al., 2021; Schwab and Lindner, 2021). This might also be an explanation for the trend in increasing NA and decreasing PA. Whereas teachers might have understood a chaotic transition from regular school operations to school lockdown and accompanying homeschooling periods during the first closure of school at t2, resignations increased, as little had changed during the second lockdown at t3.

Regarding the relation between job satisfaction and emotional experience, studies show that initial level PA predicted lower job satisfaction later at the first lockdown of schools at t2 which means that teachers who reported high PA during regular school operations reported lower job satisfaction during t2 than those who felt less PA before COVID-19. A possible explanation might be that teachers who felt more positive were more overwhelmed by the unpredicted situation than those who felt more negatively activated regarding their job.

Additionally, higher PA during the first lockdown predicted positive job satisfaction levels during the second lockdown. This finding might refer to teachers’ adjustment performance, as participants who felt more positively activated might have also been more convinced of their professional actions during the ongoing situation of the pandemic. The same applies to the investigation of NA. Teachers who felt higher levels of job satisfaction were less likely to be negatively activated during the first and second lockdowns. It is encouraging to compare these findings with the results of previous studies on the relation between job satisfaction and teachers’ emotional experience, which highlighted the significant negative correlation between high job satisfaction and negative emotional experience, as well as the significant positive correlation between high job satisfaction and PA (before COVID-19: Cheryl and Cooper, 1993; Chaplain, 1995; Ho and Au, 2006; Klassen et al., 2010; Skaalvik and Skaalvik, 2011; Toropova et al., 2021; during COVID-19: Mikušková and Verešová, 2020; Collie, 2021).

The results of the current study show that teachers successfully adjusted their professional understanding by examining their job satisfaction, but considering their emotional experience, a negative reversal could be observed. This might have occurred due to the ongoing situation of exception and the lasting psychological stress due to the enduring state of crisis.

## Limitations

Against the background of this continuing state of emergency, the current study was planned and conducted under tremendous pressure of time and urgency with the purpose of providing rapid insight into educational conditions during COVID-19. Some research limitations are the direct consequence of the urgency of the investigation.

One major limitation of the study deals with the topic of representativeness of the research. Regarding the sample and its acquisition, it must be acknowledged that forwarding an online link to an online survey does not reach the whole desired research population. In some federal states, the research team was only allowed to select data from previously chosen school types, which were selected by the local school authorities. Considering other federal states, permission was given to send the link to every school within the area of responsibility of the local school authorities. This resulted in different acquisition conditions, which led to an unequal distribution of school types.

In addition, no representativity could be achieved regarding teachers’ gender. Furthermore, as many teachers felt confronted with new challenges and demands regarding digital competencies, it can be assumed that teachers with good technical equipment and higher perceptions of self-efficacy regarding digital competences were more likely to follow the online link and fill out the questionnaire. Therefore, teachers feeling overtaxed with digital demands during homeschooling may be underrepresented within the sample.

Another aspect regarding the sample was the high dropout rate between t1/t2 and t3. When looking at the basic samples of the two points, the sample sizes are satisfyingly large, but regarding the longitudinal sample, there was a high dropout rate. Reasons may be random or due to the failure of school principals to forward the online link to their teaching staff.

It must be emphasized that in regard to the measurement points, the data considering t1 (regular school operation before COVID-19) was collected retrospectively in the process of measurement point t2 (the first lockdown). Therefore, data concerning teachers’ perceptions of job satisfaction and emotional experience might lack internal validity and causality, as they run the risk of being influenced by the actual conditions and circumstances during t2 considering the COVID-19 pandemic (see also Helm et al., 2020).

Another point that must be stressed is the diffuse understanding of the concept of job satisfaction within scientific discussions. Considering previous research and literature, different understandings and conceptualizations of job satisfaction can be observed, e.g., institutional support, perception of efficacy, satisfaction with behavior of students, etc. Therefore, it seems difficult to compare diverse studies examining teachers’ job satisfaction when relying on different definitions and concepts.

What now remains open against the background of the investigated predictors for job satisfaction and emotional experience is the question of further predicting variables of the two constructs of teachers’ characteristics. Possible variables that might be worthy of investigation are private life and home environment factors of teachers’ during homeschooling (e.g., digital resources and competences, family members such as children or the elderly who that need to be taken care of simultaneously).

## Conclusion

Approximately 1 year after the first school lockdown in Austria, the COVID-19 crisis is still ongoing. Currently, the government is intensively putting their focus on health impacts (e.g., vaccination of the population) and on economic effects (e.g., reopening restaurants, shops etc.). Socio-emotional effects of this crisis have not been addressed to the same extent. Of course, crises can also be associated with positive transformative effects. With regards to schools, some developments (e.g., increasing the use of digital tools) that are positive side effects might not just be temporary during the duration of the pandemic, but rather influence educational processes permanently. Nevertheless, some negative effects might prevail long time after the crisis. The results of the study emphasize that teachers’ well-being needs to be addressed more intensively regarding future research, practice, and measurements on institutional levels. The impact of teachers’ well-being on outcomes (e.g., burnout) as well as on students’ outcomes (e.g., academic achievement) is significant. Therefore, against the background of this study’s results, it seems important to address the remarkable negative change in teachers’ job satisfaction and emotional experiences to avoid long-term consequential damage on individuals (teachers, students) and institutions (schools, educational system).

## Data Availability Statement

The raw data supporting the conclusions of this article will be made available by the authors, without undue reservation.

## Ethics Statement

The studies involving human participants were reviewed and approved by local school boards in Austria. The patients/participants provided their written informed consent to participate in this study.

## Author Contributions

K-TL: conception and design of study, organized the database, first draft of the manuscript, wrote sections of the manuscript, and incorporation of all comments of authors. HS: conception and design of study, performed the statistical analysis, and wrote sections of the manuscript. SS: conception and design of study and wrote sections of the manuscript. All authors contributed to manuscript revision, read, and approved the submitted version.

## Conflict of Interest

The authors declare that the research was conducted in the absence of any commercial or financial relationships that could be construed as a potential conflict of interest.

## Publisher’s Note

All claims expressed in this article are solely those of the authors and do not necessarily represent those of their affiliated organizations, or those of the publisher, the editors and the reviewers. Any product that may be evaluated in this article, or claim that may be made by its manufacturer, is not guaranteed or endorsed by the publisher.

## Footnotes

1 Schwab, S., Gutschik, A., Lindner, K.-T., and Kast, J. (submitted). Inklusive Bildung in Zeiten der Corona Krise – Empirische Einblicke in das emotionale Erleben von Lehrkräften, Schülerinnen und Eltern [Inclusive Education in Times of Corona Crisis - Empirical Insights into the Emotional Experiences of Teachers, Students, and Parents].

2 Lindner, K.-T., Gitschthaler, M., Gutschik, A., Corazza, R., and Schwab, S. (submitted). Increased Educational Disadvantage of Refugee Students in German Language Support Classes during COVID-19 School Closures in Austria – Perceptions and Pedagogical Reactions of Austrian Teachers.

3 This negative effect might be real or indicate a suppressor effect, as the initial correlation between these variables was non-significant (*r* = 0.05) and the prediction between t1 and t2 job satisfaction was Std.Beta = 0.46, while their correlation was *r* = 0.39.
